# Supplementary figures and images for: Duvernoy’s Gland Transcriptomics of the Plains Black-Headed Snake, Tantilla nigriceps (Squamata, Colubridae): Unearthing the Venom of Small Rear-Fanged Snakes
Source: Toxins (Basel). 2021 May 6;13(5):336. doi: 10.3390/toxins13050336 (PMC8148590; doi:10.3390/toxins13050336)

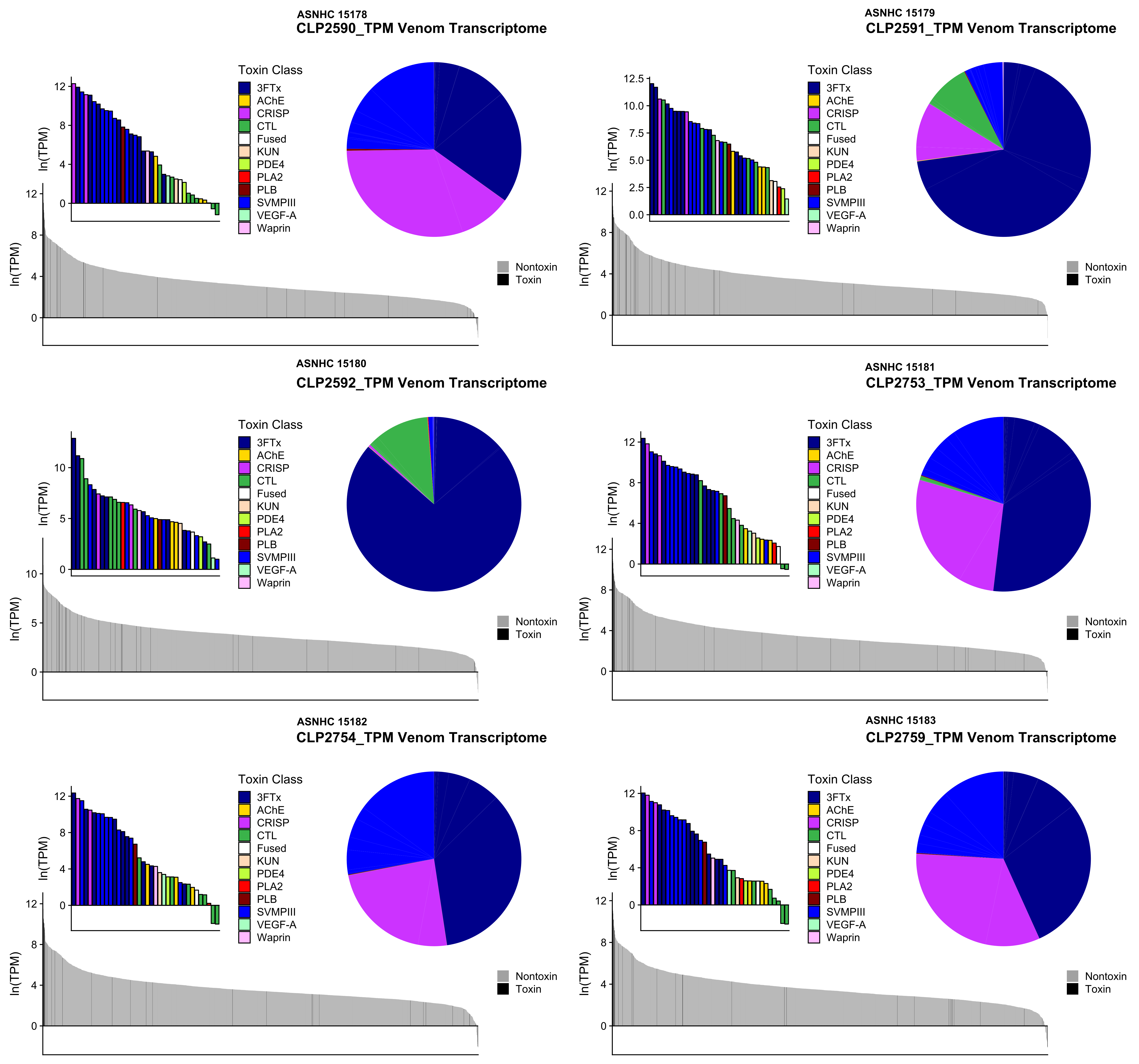

Supplement: Supplementary file 1 [file toxins-13-00336-s001.zip › FigureS1-individualFigs.png]
